# Effects of perturbations on the stability of equilibrium points in the CR3BP with luminous and heterogeneous spheroid primaries

**DOI:** 10.1038/s41598-022-06328-y

**Published:** 2022-02-18

**Authors:** Jagadish Singh, Shitu Muktar Ahmad

**Affiliations:** Department of Mathematics, Faculty of Physical Science, Ahmadu Bello University, Zaria, Nigeria

**Keywords:** Astronomy and planetary science, Mathematics and computing, Physics

## Abstract

This paper studies the position and stability of equilibrium points in the circular restricted three-body problem under the influence of small perturbations in the Coriolis and centrifugal forces when the primaries are radiating and heterogeneous oblate spheroids. It is seen that there exist five libration points as in the classical restricted three-body problem, three collinear $L_{i} ,(i = 1,2,3)$ and two triangular $L_{i} ,(i = 4,5)$. It is also seen that the triangular points are no longer to form equilateral triangles with the primaries rather they form simple triangles with line joining the primaries. It is further observed that despite all perturbations the collinear points remain unstable while the triangular points are stable for $0 < \mu  < \mu _{c}$ and unstable for $\mu _{c}  \le \mu  \le \frac{1}{2}$, where $\mu _{c}$ is the critical mass ratio depending upon aforementioned parameters. It is marked that small perturbation in the Coriolis force, radiation and heterogeneous oblateness of the both primaries have destabilizing tendencies. Their numerical examination is also performed.

## Introduction

The restricted three-body problem (R3BP) deals with the motion of an infinitesimal particle under the Newtonian gravitational attraction of two bodies, called primaries. It is known that in the planar general three bodies attracting each other according to Newtonian gravitational law, there exist only two permanent central configurations, namely the collinear (Eulerian) and the equilateral (Lagrangian). In the first case, the primary bodies of the problem lie on a single straight line while in the second one, the primary bodies lie at the vertices of an equilateral triangle. The restricted three- body problem possesses five equilibrium points $L_{i} ,(i = 1,2,3,4,5)$: three collinear points $L_{1} ,L_{2} ,L_{3}$ and two noncollinear (triangular) points $L_{4} ,L_{5}$. The collinear points are unstable for 0 ≤ µ ≤ ½ and the triangular points are stable for the mass ratio µ < 0.03852…^1^. He considered the effect of a small perturbation in the Coriolis force on the stability of the equilibrium points keeping the centrifugal force constant, He found that the collinear points are still unstable but the triangular points bear a relation between the critical value of the mass parameter µ_c_ and the change $\epsilon$ in the Coriolis force: ${\upmu }_{{\text{c}}} = {\upmu }_{0} + \frac{16\epsilon }{{3\sqrt {69} }}$ implying that the Coriolis force is a stabilizing force^2^. Studied the effect of small perturbations in the Coriolis and centrifugal forces on the stability of equilibrium points in the restricted problem. They observed that the collinear points remain collinear, but the triangular libration points nearly form equilateral triangles with the primaries, they also revealed that these perturbations have null effects on the stability of collinear points, but have considerate effects on the stability of triangular points. The R3BP with disturbing agents (such as Oblateness, Radiation pressure, Coriolis and centrifugal forces, the Pointing-Robertson and Yarkovsky effects, the atmospheric drag and solar wind) was investigated by several scientists. Various researchers^2–5^ considered small perturbations in the Coriolis and centrifugal forces and described their effects on the motion of the third body^6^. Examined the equilibrium points in the perturbed restricted three body problem with triaxial and luminous primaries. He observed that the position of the five libration points are affected by the radiation, triaxiality and a small perturbation in the centrifugal force, but not affected by that of Coriolis force. He also found that the Coriolis force has a stabilizing tendency, while the centrifugal force, radiation and oblateness of the primary body have destabilizing effects^7^. Deals with the stationary solution of the planar restricted three body problem when the primaries are heterogeneous oblate spheroid with three layers of different density and source of radiation. They showed that the triangular equilibrium points $L_{4,5}$ are stable for $0 < \mu < \mu_{c}$, andunstable for $\mu_{c} \le \mu \le \frac{1}{2}$, where $\mu_{c}$ is the critical mass parameter influenced by parameters involved^8^. Studied the circular restricted three body problem when the both primaries are heterogeneous spheroid of three layers of different densities and the infinitesimal body varies its mass. They locate all the equilibrium points of both the in-plane and out-of-plane and proved that they are unstable^9^. Investigated the non linear stability of the triangular libration point *L*_*4*_ in the restricted three body problem when the smaller primary is a heterogeneous spheroid. They observed that the positions of the non collinear equilibrium point *L*_*4*_ is significantly affected by the heterogeneous oblate spheroid with three layers having different densities and this point is stable in the nonlinear hence in the range of linear stability except at three points at which KAM Theorem fails.

In this paper, our aim is to study the effects of small perturbations in the Coriolis and centrifugal forces when the both primaries are heterogeneous oblate spheroids and source of radiation pressure. This research paper is organized as follows: in “Introduction” Section: we have reviewed the literature. In Equations of motion Section: the equations governing the motion of infinitesimal body are presented. “Location of equilibrium points” Section: describes the position of equilibrium points. “Stability of the libration points” Section: we have analyzed the stability of the libration equilibrium points, while discussion and conclusion are given in “Discussion” Section and “Conclusion” Section, respectively.

## Equations of motion

We consider the primaries of masses $m_{1}$ and *m*_2_ $(m_{1} > m_{2} )$ as heterogeneous spheroid with three layers of different densities, source of radiation and we assume that the infinitesimal mass $m$ is moving under the gravitational attraction of the said primaries. Using dimensionless variables, the equations of motion of the infinitesimal body in a synodic coordinate system can be written as^3,7^:

1 $$ \begin{gathered} \ddot{x} - 2n\alpha \dot{y} = \Omega_{x} \hfill \\ \ddot{y} + 2n\alpha \dot{x} = \Omega_{y} \hfill \\ \end{gathered} $$

where

2 $$ \Omega = \frac{{n^{2} \beta }}{2}\left( {x^{2} + y^{2} } \right) + q_{1} \left( {\frac{1 - \mu }{{r_{1} }} + \frac{{k_{1} }}{{2r_{1}^{3} }}} \right) + q_{2} \left( {\frac{\mu }{{r_{2} }} + \frac{{k_{2} }}{{2r_{2}^{3} }}} \right), $$

and $\Omega_{x}$, denote the partial derivative of $\Omega$ with respect to $x$ and $y$ respectively.

3 $$ \begin{gathered} r_{1}^{2} = \left( {x - \mu } \right)^{2} + y^{2} . \hfill \\ r_{2}^{2} = \left( {x - \mu + 1} \right)^{2} + y^{2} . \hfill \\ \end{gathered} $$

$$ k_{j} = \frac{4\pi }{3}\sum\limits_{i = 1}^{3} {\left( {\delta_{i}^{(j)} - \delta_{i + 1}^{(j)} } \right)(A_{i}^{(j)} )^{2} C_{i}^{(j)} \sigma_{i}^{(j)} \;(j = 1,2)} . $$

$$ \delta_{i}^{(j)} = \frac{{\rho_{i}^{(j)} }}{M},\;A_{i}^{(j)} = \frac{{a_{i}^{(j)} }}{R},\;C_{i}^{(j)} = \frac{{c_{i}^{(j)} }}{R}\;(i = 1,2,3) $$

$$ \sigma_{i}^{(j)} = \frac{{(a_{i}^{(j)} )^{2} - (c_{i}^{(j)} )^{2} }}{5},\;\rho_{4}^{(j)} = 0,\;k_{j} < < 1 $$

*M* = *m*_*1*_ + *m*_*2*_

$$ \sigma_{i}^{(j)} = {\text{Oblateness factor}} $$

$$ a_{i} ,c_{i} = {\text{The semi}} - {\text{axes of the heterogeneous spheroid}} $$

R = dimensional distance between the primaries.

The mean motion n of the primaries is

$$ n^{2} = 1 + \frac{{3k_{3} }}{2} $$

where

$$ k_{3} = \frac{{\sum\limits_{i = 1}^{3} {(\rho_{i}^{(1)} \rho_{i}^{(2)} - \rho_{i + 1}^{(1)} \rho_{i + 1}^{(2)} )a_{i}^{(1)2} c_{i}^{(!)} (a_{i}^{(2)} )^{2} c_{i}^{(2)} (\sigma_{i}^{(1)} + \sigma_{i}^{(2)} )} }}{{\sum\limits_{i = 1}^{3} {(\rho_{i}^{(1)} - \rho_{i + 1}^{(1)} )(a_{i}^{(1)} )^{2} c_{i}^{(1)} \sum\limits_{i = 1}^{3} {(\rho_{i}^{(2)} - \rho_{i + 1}^{(2)} )(a_{i}^{(2)} )^{2} c_{i}^{(2)} } } }},\quad k_{3} < < 1, $$

here $$r_{1}$$ and $$r_{2}$$ are distances of the infinitesimal body from the primaries, $$\rho_{i}$$ are the densities for the layers, *q*_*1*_ and *q*_*2*_ are the radiation factors of the primaries. The resultant force acting on the infinitesimal body is $$F_{i} =$$ $$Fg_{i} (1 - \frac{{Fp_{i} }}{{Fg_{i} }}) = Fg_{i} (1 - \delta_{i} )$$ where $$\delta_{i} = \frac{{Fp_{i} }}{{Fg_{i} }},$$$$0 < \delta_{i} < < 1$$ and $$q_{i} = 1 - \delta_{i} ,(i = 1,2)$$ $$Fg_{i}$$ and $$Fp_{i}$$ are gravitational and radiation pressure forces respectively^6^, $$\alpha = 1 + \epsilon$$, $$\left| \epsilon \right| < < 1$$ and $$\beta = 1 + \epsilon ^{\prime},$$ $$\left| {\epsilon ^{\prime}} \right| < < 1$$ are the parameters for the Coriolis and centrifugal forces respectively for which $$\epsilon$$ and $$\epsilon ^{\prime}$$ are small perturbations.

From Eq. (1) we obtain Jacobi integral as

4 $$ \dot{x}^{2} + \dot{y}^{2} - 2\Omega + C = 0. $$

where $\dot{x}$ and $\dot{y}$ are the velocity components and C is the Jacobian constant.

## Location of equilibrium points

The position of equilibrium points can be found by setting $\dot{x} = \dot{y} = \ddot{x} = \ddot{y} = 0$ in the equations of motion.

### Location of triangular points

The triangular points are the solutions of equations $$\Omega_{x} = 0,$$$$\Omega_{y} = 0$$ with $$y \ne 0$$, therefore from Eqs. (2) and (3) we have

5 $$ \begin{aligned} & n^{2} \beta x - \frac{{\left( {1 - \mu } \right)\left( {x - \mu } \right)q_{1} }}{{r_{1}^{3} }} - \frac{{3\left( {x - \mu } \right)k_{1} q_{1} }}{{2r_{1}^{5} }} - \frac{{\mu \left( {x - \mu + 1} \right)q_{2} }}{{r_{2}^{3} }} - \frac{{3\left( {x - \mu + 1} \right)k_{2} q_{2} }}{{2r_{2}^{5} }} = 0, \\ & n^{2} \beta y - \frac{{(1 - \mu )yq_{1} }}{{r_{1}^{3} }} - \frac{{3yk_{1} q_{1} }}{{2r_{1}^{5} }} - \frac{{\mu yq_{2} }}{{r_{2}^{3} }} - \frac{{3yk_{2} q_{2} }}{{2r_{2}^{5} }} = 0. \\ \end{aligned} $$

When the primaries are neither radiating nor heterogeneous spheroids ($$k_{1} = k_{2} = k_{3} = 0$$, $$q_{1} = q_{2} = 1,\;n^{2} = 1$$), the solutions of Eq. (5) reduced to $$r_{1} = r_{2} = \beta^{{ - {\raise0.7ex\hbox{$1$} \!\mathord{\left/ {\vphantom {1 3}}\right.\kern-\nulldelimiterspace} \!\lower0.7ex\hbox{$3$}}}}$$. We suppose that when the primaries are radiating and heterogeneous spheroid, the value of the solutions equations $$r_{1}$$ and $$r_{2}$$ are

6 $$ \begin{gathered} r_{1} = \beta^{{ - {\raise0.7ex\hbox{$1$} \!\mathord{\left/ {\vphantom {1 3}}\right.\kern-\nulldelimiterspace} \!\lower0.7ex\hbox{$3$}}}} + \varepsilon_{1} \hfill \\ r_{2} = \beta^{{ - {\raise0.7ex\hbox{$1$} \!\mathord{\left/ {\vphantom {1 3}}\right.\kern-\nulldelimiterspace} \!\lower0.7ex\hbox{$3$}}}} + \varepsilon_{2} \hfill \\ \end{gathered} $$

where $\varepsilon_{1} ,\varepsilon_{2} < < 1$ are very small.

Putting these values of $r_{1} ,r_{2}$ into system (3) and neglecting the second and highest order terms in $\varepsilon_{1}$, $\varepsilon_{2}$, we have

$$ x = \mu - \frac{1}{2} - \varepsilon_{1} \beta^{{ - {\raise0.7ex\hbox{$1$} \!\mathord{\left/ {\vphantom {1 3}}\right.\kern-\nulldelimiterspace} \!\lower0.7ex\hbox{$3$}}}} + \varepsilon_{2} \beta^{{ - {\raise0.7ex\hbox{$1$} \!\mathord{\left/ {\vphantom {1 3}}\right.\kern-\nulldelimiterspace} \!\lower0.7ex\hbox{$3$}}}} $$

7 $$ y^{2} = \beta^{{ - {\raise0.7ex\hbox{$2$} \!\mathord{\left/ {\vphantom {2 3}}\right.\kern-\nulldelimiterspace} \!\lower0.7ex\hbox{$3$}}}} - \frac{1}{4} + \varepsilon_{1} \beta^{{ - {\raise0.7ex\hbox{$1$} \!\mathord{\left/ {\vphantom {1 3}}\right.\kern-\nulldelimiterspace} \!\lower0.7ex\hbox{$3$}}}} + \varepsilon_{2} \beta^{{ - {\raise0.7ex\hbox{$1$} \!\mathord{\left/ {\vphantom {1 3}}\right.\kern-\nulldelimiterspace} \!\lower0.7ex\hbox{$3$}}}} $$

Substituting the values of $$r_{1} ,r_{2}$$ from Eqs. (6) and $$x,y$$ from Eqs. (7) into Eqs. (5) and using $$\alpha = 1 + \epsilon$$, $$\beta = 1 + \epsilon ^{\prime}$$, $$q_{i} = 1 - \delta_{i}$$ and restricting ourselves to only linear terms in $$ \epsilon ,\;\epsilon ^{\prime }  $$$$\delta_{1} ,\delta_{2} ,k_{1} ,k_{2} ,k_{3} ,\varepsilon_{1}$$ and $$\varepsilon_{2} ,$$ we obtain

8 $$ \begin{gathered} \varepsilon_{1} = \frac{{k_{1} }}{{2\left( {1 - \mu } \right)}} - \frac{{k_{3} }}{2} - \frac{{\delta_{1} }}{3} \hfill \\ \varepsilon_{2} = \frac{{k_{2} }}{2\mu } - \frac{{k_{3} }}{2} - \frac{{\delta_{2} }}{3} \hfill \\ \end{gathered} $$

Putting these values of Eqs. (8) into Eqs. (7) then we get

9 $$ \begin{aligned} & x = \mu - \frac{1}{2} + \frac{1}{2}\left( {\frac{{k_{2} }}{\mu } - \frac{{k_{1} }}{1 - \mu }} \right) + \frac{1}{3}\left( {\delta_{1} - \delta_{2} } \right), \\ & y = \pm \frac{\sqrt 3 }{2}\left[ {1 - \frac{4}{9}\epsilon ^{\prime} + \frac{1}{3}\left( {\frac{{k_{1} }}{1 - \mu } + \frac{{k_{2} }}{\mu }} \right) - \frac{2}{3}k_{3} \left( {\delta_{1} + \delta_{2} } \right)} \right] \\ \end{aligned} $$

**Figure 1:**
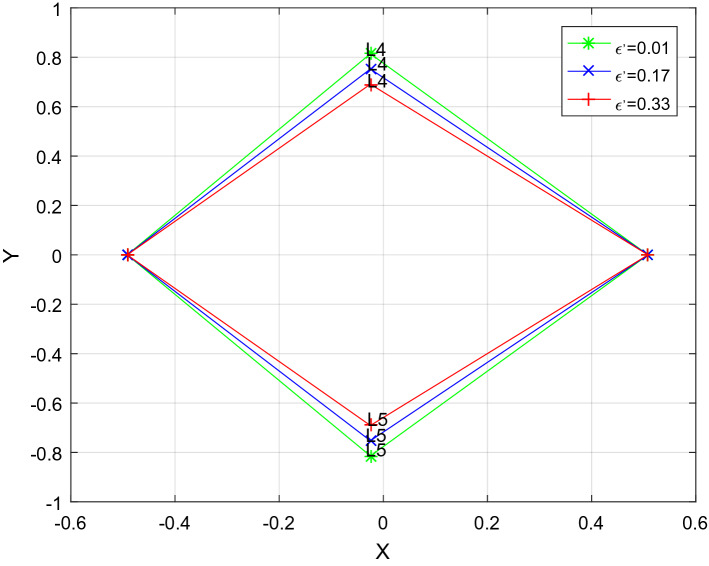
Effect of a small perturbation in the centrifugal force on the location of triangular points for $$\delta_{1}$$ = 0.10*, *$$\delta_{2}$$ = 0.15 $$k_{1} = 1.58302 \times 10^{ - 7} ,k_{2} = 9.83933 \times 10^{ - 18} ,k_{3} = 3.13153 \times 10^{ - 8}$$$$\mu = 0.4918$$.

**Figure 2:**
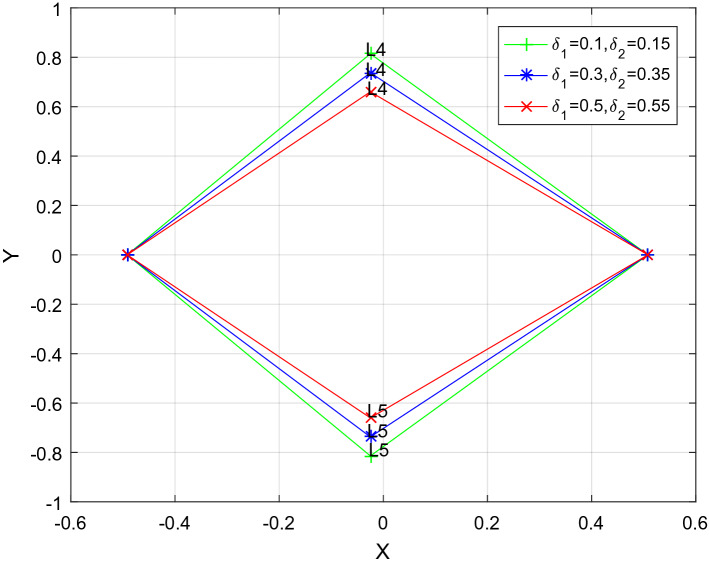
Effect of radiation of the both primaries on the location of triangular points for $\varepsilon^{\prime}$ = *0.01* $k_{1} = 1.58302 \times 10^{ - 7} ,k_{2} = 9.83933 \times 10^{ - 18} ,k_{3} = 3.13153 \times 10^{ - 8}$, $\mu = 0.4918$.

Thus the coordinates $\left( {x, \pm y} \right)$ denoted by $L_{4}$ and $L_{5}$ are known as triangular points. One can observe that the locations of the triangular points depend on the small perturbation in the centrifugal force, heterogeneous oblateness and radiation of both primaries. We now compute the position of triangular points for the binary system Upsilon^4^Eridani numerically. The radiation pressure force $q_{i} = 1 - \delta_{i}$ $i = 1,2$ ^10^.Taking $k = 1$, on the basis of Stefan-Boltzmann's law,$q = 1 - \frac{AkL}{{r\rho M}}$, where M and L are the mass and luminosity of a star respectively; r and $\rho$ are the radius and density of moving body; k is the radiation pressure efficiency factor of star; In the CGS system,$A = 2.9838\; \times \;10^{ - 5}$_*,*_ Suppose $r = 2 \times 10^{ - 2} \;{\text{cm}}\;{\text{and}}\;\rho = 1.4\;{\text{g}}\;{\text{cm}}^{ - 3}$. The relevant numerical data of the system obtain from Wikipedia are given in Tables 1, 2, 3, 4 and 5 presented the effect of radiation pressure of the primaries and a small perturbation in centrifugal force on the position of triangular points with and/or without heterogeneous oblate spheroid of the binary system. Figures 1 and 2 shows the triangular points move closer to the *x*-axis as observed by the effects of perturbations.

**Table 1:** Relevant numerical data.

Binary system	Masses (Msun)		Luminosity (Lsun)		Eccentricity	Mass ratio	Radiation pressure	
	M_1_	M_2_	L_1_	L_2_	(e)	$\mu$	$q_{1}$	$q_{2}$
Upsilon^4^Eridani	3.24	3.14	104.9	90.7	0.00	0.4918	0.9339	0.9411

**Table 2:** Location of the triangular equilibrium points for $\mu = 0.4918$, $k_{1} = k_{2} = k_{3} = 0$.

$\delta_{1}$	$\delta_{2}$	$\epsilon ^{\prime}$	$x$	± *y*
0	0	0	− 0.00820	0.86603
0.06	0	0	0.01180	0.85448
0.08	0	0	0.01847	0.85063
0	0.05	0	− 0.02487	0.85640
0	0.07	0	− 0.03153	0.85255
0	0	0.01	− 0.00820	0.86218
0	0	0.02	− 0.00820	0.85833
0.06	0.05	0.01	− 0.00487	0.84101
0.8	0.07	0.02	− 0.00487	0.82946

**Table 3:** Effect of radition of the bigger primary on the location of triangular equilibrium points for $$k_{1} = 1.58302 \times 10^{ - 7} ,k_{2} = 9.83933 \times 10^{ - 18} ,k_{3} = 3.13153 \times 10^{ - 8}$$$$\mu = 0.4918$$.

$\delta_{1}$	$\delta_{2}$	$\epsilon ^{\prime}$	*x*	± *y*
0.06	0.05	0.01	− 0.00487	0.84101
0.08	0.05	0.01	0.01799	0.83716
0.10	0.05	0.01	0.00847	0.83331
0.30	0.05	0.01	0.07513	0.79482
0.50	0.05	0.01	0.14180	0.75633
0.70	0.05	0.01	0.20847	0.71784
0.90	0.05	0.01	0.27513	0.67935

**Table 4:** Effect of radiation of the smaller primary on the location of triangular equilibrim points for $$k_{1} = 1.58302 \times 10^{ - 7} ,k_{2} = 9.83933 \times 10^{ - 18} ,k_{3} = 3.13153 \times 10^{ - 8}$$$$\mu = 0.4918$$.

$\delta_{1}$	$\delta_{2}$	$\epsilon ^{\prime}$	*x*	± *y*
0.06	0.05	0.01	− 0.00487	0.84101
0.06	0.07	0.01	− 0.01153	0.83716
0.06	0.09	0.01	− 0.01820	0.83309
0.06	0.20	0.01	− 0.05487	0.81214
0.06	0.40	0.01	− 0.12153	0.77365
0.06	0.60	0.01	− 0.18820	0.73516
0.06	0.80	0.01	− 0.25487	0.69667

**Table 5:** Effect of a small perturbation in centrifugal force on the location of triangular equilibrium points for $$k_{1} = 1.58302 \times 10^{ - 7} ,k_{2} = 9.83933 \times 10^{ - 18} ,k_{3} = 3.13153 \times 10^{ - 8}$$$$\mu = 0.4918$$.

$\delta_{1}$	$\delta_{2}$	$\epsilon ^{\prime}$	$x$	± *y*
0.06	0.05	0.01	− 0.00487	0.84101
0.06	0.05	0.02	− 0.00487	0.83716
0.06	0.05	0.03	− 0.00487	0.83331
0.06	0.05	0.04	− 0.00487	0.82946
0.06	0.05	0.05	− 0.00487	0.82561
0.06	0.05	0.06	− 0.00487	0.82176
0.06	0.05	0.07	− 0.00487	0.81791

### Location of the collinear points

The positions of collinear points are the solutions of the equations.

$$\Omega_{x} = 0,\;\Omega_{y} = 0,\;y = 0$$,

It shows that the collinear points lie on the $x$-*axis .*Substituting $y = 0$ in the left hand side of the first equation of system (5) and denoting the resulting expression by $f(x)$, we have

10 $$ f(x) = n^{2} \beta x - \frac{{\left( {1 - \mu } \right)\left( {x - \mu } \right)q_{1} }}{{r_{1}^{3} }} - \frac{{3\left( {x - \mu } \right)k_{1} q_{1} }}{{2r_{1}^{5} }} - \frac{{\mu \left( {x - \mu + 1} \right)q_{2} }}{{r_{2}^{3} }} - \frac{{3\left( {x - \mu + 1} \right)k_{2} q_{2} }}{{2r_{2}^{5} }} = 0 $$

where $$r_{1} = \left| {x - \mu } \right|\mathop {}\limits^{{}} and\mathop {}\limits^{{}} r_{2} = \left| {x - \mu + 1} \right|$$

To locate the collinear equilibrium points, we divide the orbital plane into three parts: $- \infty < x < \mu - 1,\mu - 1 < x < \mu$, and $\mu < x < \infty$ with respect to the primaries, there exists a collinear point $L_{i} (i = 1,2,3)$, in each of the interval.

*Case 1* $$L_{1} ( - \infty x < \mu - 1)$$

Let the collinear point $L_{1}$ be on the left-hand side of the smaller primary at a distance *h*_*1*_ from it on the $x$-axis. Then.

Now putting, $$x = \mu - 1 - h_{1}$$, $$x - \mu = - \left( {1 + h_{1} } \right) = r_{1}$$.

Since the distance between the primaries is unity, substituting all the above values in Eq. (10), we have

11 $$ \begin{aligned}    &  - 2n^{2} \beta h_{1}^{9}  - 2n^{2} \beta \left( {4 + \left( {1 - \mu } \right)} \right)h_{1}^{8}  - 2n^{2} \beta \left( {6 + 4\left( {1 - \mu } \right)} \right)h_{1}^{7}  \\     &  - 2\left( {n^{2} \beta \left( {4 + 6\left( {1 - \mu } \right)} \right) - \left( {1 - \mu } \right)q_{1}  - \mu q_{2} } \right)h_{1}^{6}  - 2\left( {n^{2} \beta \left( {1 + 4\left( {1 - \mu } \right)} \right) - 2\left( {1 - \mu } \right)q_{1}  - 4\mu q_{2} } \right)h_{1}^{5}  \\     &  - 2\left( {n^{2} \beta \left( {1 - \mu } \right) - \left( {1 - \mu } \right)q_{1}  - \frac{{3k_{1} q_{1} }}{2} - 6\mu q_{2}  - \frac{{3k_{2} q_{2} }}{2}} \right)h_{1}^{4}  + 2\left( {(4\mu  + 6k_{2} )q_{2} } \right)h_{1}^{3}  \\     &  + 2\left( {(\mu  + 9k_{2} )q_{2} } \right)h_{1}^{2}  + 12k_{2} q_{2} h_{1}  + 3k_{2} q_{2}  = 0 \\  \end{aligned}  $$

This is an algebraic polynomial equation of degree nine in $h_{1}$ and there is only one change of sign, therefore, there exists at least one real root in it. The collinear point .$L_{1}$.located by $x_{1} = \mu - 1 - h_{1}$.

*Case 2* $$L_{2} (\mu - 1 < x < \mu )$$.

Let the collinear point $L_{2}$ be on the right-hand side of the smaller primary at a distance *h*_*2*_ from it on the $x$-axis.

We put $$x = \mu - 1 + h_{2} ,$$$$x - \mu = - 1 + h_{2} = r_{1} ,$$

Now, substituting the values of $r_{1} ,r_{2} ,x$ in to Eq. (10), we have

12 $$ \begin{gathered} 2n^{2} \beta h_{2}^{9} - 2n^{2} \beta \left( {4 + \left( {1 - \mu } \right)} \right)h_{2}^{8} + 2n^{2} \beta \left( {6 + 4\left( {1 - \mu } \right)} \right)h_{2}^{7} - 2\left( {n^{2} \beta \left( {4 + 6\left( {1 - \mu } \right)} \right) - \left( {1 - \mu } \right)q_{1} + \mu q_{2} } \right)h_{2}^{6} \hfill \\ + 2\left( {n^{2} \beta \left( {1 + 4\left( {1 - \mu } \right)} \right) - 2\left( {1 - \mu } \right)q_{1} + 4\mu q_{2} } \right)h_{2}^{5} - 2\left( {n^{2} \beta \left( {1 - \mu } \right) - \left( {1 - \mu } \right)q_{1} - \frac{{3k_{1} q_{1} }}{2} + \left( {6\mu + \frac{{3k_{2} }}{2}} \right)q_{2} } \right)h_{2}^{4} \hfill \\ + 2\left( {4\mu + 6k_{2} } \right)q_{2} h_{2}^{3} - 2\left( {\mu - 9k_{2} } \right)q_{2} h_{2}^{2} + 12k_{2} q_{2} h_{2} - 3k_{2} q_{2} = 0 \hfill \\ \end{gathered} $$

*Case 3* $$L_{3} (\mu < x < \infty )$$.

Let the collinear point $L_{3}$ be on the right-hand side of the bigger primary at a distance $h_{3}$ from it on the $x$-axis.

Putting,$x = \mu + h_{3}$, $x - \mu = h_{3} = r_{1}$ and substituting the values of $r_{1} ,r_{2}$ and $x$ into Eq. (10),we get

13 $$ \begin{aligned}    & 2n^{2} \beta h_{3}^{9}  + 2n^{2} \beta \left( {4 + \mu } \right)h_{3}^{8}  + 4n^{2} \beta \left( {3 + 2\mu } \right)h_{3}^{7}  + 2\left( {2n^{2} \beta \left( {2 + 3\mu } \right) - \left( {1 - \mu } \right)q_{1}  - \mu q_{2} } \right)h_{3}^{6}  \\     &  + 2\left( {n^{2} \beta \left( {1 + 4\mu } \right) - 4\left( {1 - \mu } \right)q_{1}  - 2\mu q_{2} } \right)h_{3}^{5}  + 2\left( {n^{2} \beta \mu  - 6\left( {1 - \mu } \right)q_{1}  - \frac{{3k_{1} q_{1} }}{2} - \left( {\mu  + \frac{{3k_{2} }}{2}} \right)q_{2} } \right)h_{3}^{4}  \\     &  - 4\left( {2\left( {1 - \mu } \right)q_{1}  + 3k_{1} q_{1} } \right)h_{3}^{3}  - 2\left( {\left( {1 - \mu } \right)q_{1}  + 9k_{1} q_{1} } \right)h_{3}^{2}  - 12k_{1} q_{1} h_{3}  - 3k_{1} q_{1}  = 0 \\  \end{aligned}  $$

**Figure 3:**
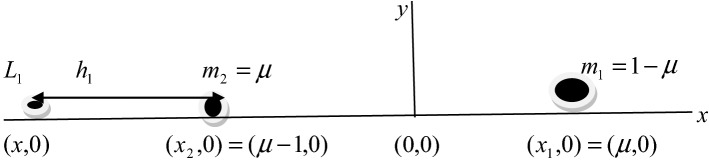
Position of the collinear equilibrium point $L_{1}$.

**Figure 4:**
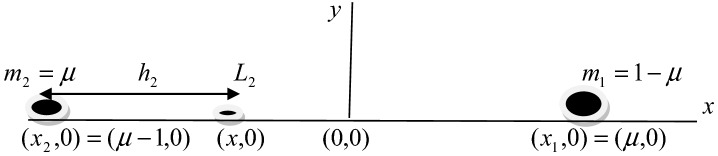
Position of the collinear equilibrium point $L_{2}$.

**Figure 5:**
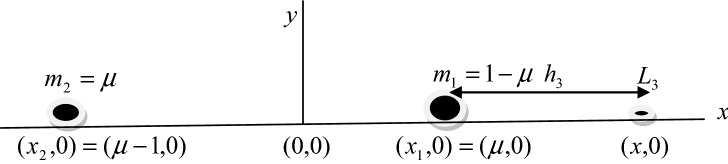
Position of the collinear equilibrium point $L_{3}$.

Equations (11), (12) and (13) are ninth degree equations and there is only one positive real root in each case. This is physically acceptable root corresponds to one of the three collinear points $Li(i = 1,2,3)$. The Figs. 3, 4 and 5 are the disposition of the collinear equilibrium points in which the Figs. 3 and 5 describe the collinear points $L_{1}$ and $L_{3}$ moves closer to the primaries and the collinear point $L_{2}$ is located between the primaries in Fig. 4.

Following^11^, we compute the roots of the algebraic polynomial equations corresponding to the collinear points *Li* (*i* = 1, 2, 3) numerically, with the help of Mathematica (wolfram2010) and Matlap (R2007b) software packages. The binary system Upsilon^4^Eridani portrayed *µ* = *0.4918, q*_*1*_ = 0.9339*, q*_*2*_ = 0.9411*,*$$\beta = 1 +$$$$\epsilon ^{\prime}$$*,*$$\epsilon ^{\prime} = 0.01$$*, k*_*1*_ = 1.58302 × 10^–7^*, k*_*2*_ = 9.83933 × 10^–18^*, k*_*3*_ = 3.13153 × 10^–8^. We obtain the coordinate of the collinear equilibrium points for different cases as classified in the following order while the effect of distances from primaries and the positions of the collinear points for different cases shown in Table 6.

(i) Absence of radiation pressure of the both primaries, small perturbations in the Coriolis and centrifugal forces and heterogeneous oblate spheroid (classical case)
(ii) A small perturbation in the centrifugal force with heterogeneous oblate spheroid only
(iii) Heterogeneous oblate spheroid only
(iv) Radiation pressure of the bigger primary with heterogeneous oblate spheroid only
(v) The radiation pressure of the both primaries only
(vi) Radiation pressure of the smaller primary with heterogeneous oblate spheroid only
(vii) Presence of radiation pressure of the both primaries, small perturbations in the Coriolis and centrifugal forces and heterogeneous oblate spheroids

**Table 6:** Effect of the cases on the coordinates of collinear libration points of L*i* (*i* = *1,2,3*).

Case	*h*_*1*_*(L*_*1*_*)*	*L*_*1*_	*h*_*2*_*(L*_*2*_*)*	*L*_*2*_	*h*_*3*_*(L*_*3*_*)*	*L*_*3*_
1	0.69308	− 1.20124	0.49664	− 0.01158	0.70379	1.19555
2	0.69017	− 1.19837	0.49663	− 0.01167	0.70086	1.19266
3	0.69304	− 1.20135	0.49662	− 0.01176	0.70375	1.19563
4	0.69023	− 1.19843	0.50468	− 0.00353	0.68690	1.17871
5	0.67542	− 1.18362	0.49751	− 0.01063	0.68444	1.17624
6	0.67827	− 1.18647	0.48952	− 0.01868	0.70133	1.19313
7	0.67263	− 1.18083	0.49758	− 0.01071	0.68163	1.17343

## Stability of the libration points

We substitute $x = a + \xi$ and $y = b + \eta$ in the equations of motion (1), where $\xi ,\eta$ are small displacements of the infinitesimal body and $(a,b)$ are the coordinates of the libration equilibrium point under consideration. Then we obtain the variational equations as

14 $$ \begin{gathered} \ddot{\xi } - 2n\alpha \dot{\eta } = \xi \Omega_{xx}^{0} + \eta \Omega_{xy}^{0} \hfill \\ \ddot{\eta } + 2n\alpha \dot{\xi } = \xi \Omega_{yx}^{0} + \eta \Omega_{yy}^{0} \hfill \\ \end{gathered} $$

here the linear terms in $\xi$ and $\eta$, are only considered. The second order partial derivatives of $\Omega$ are denoted by subscripts, and the superscript “0” indicates that the derivatives are to be evaluated at the libration equilibrium point $(a,b)$.

The corresponding characteristic equation of the variational Eqs. (14) can be written as

15 $$ \lambda^{4} + \left( {4n^{2} \alpha^{2} - \Omega_{xx}^{0} - \Omega_{yy}^{0} } \right)\lambda^{2} + \Omega_{xx}^{0} \Omega_{yy}^{0} - \left( {\Omega_{xy}^{0} } \right)^{2} = 0, $$

### Stability of triangular points

For the triangular points, we have computed the second partial derivative of $\Omega$ occurred in the Eq. (14), and they are

$$ \Omega_{xx}^{0} = \frac{3}{4} + \frac{5}{4}\epsilon ^{\prime} + \frac{3}{8}\left( {1 + \frac{3}{1 - \mu } - \frac{7\mu }{{1 - \mu }}} \right)k_{1} + 3\left( {1 - \frac{1}{2\mu }} \right)k_{2} + \frac{15}{8}k_{3} - \frac{1}{2}\left( {1 - 3\mu } \right)\delta_{1} + \frac{1}{2}\left( {2 - 3\mu } \right)\delta_{2} , $$

$$ \begin{aligned} \Omega_{xy}^{0} = \Omega_{yx}^{0} = & - \frac{3\sqrt 3 }{4}\left( {1 - 2\mu } \right) - \frac{11\sqrt 3 }{{12}}\left( {1 - 2\mu } \right)\epsilon ^{\prime} - \frac{\sqrt 3 }{8}\left( {15 - \frac{7}{1 - \mu } + \frac{11\mu }{{1 - \mu }}} \right)k_{1} + \frac{\sqrt 3 }{2}\left( {1 + \frac{1}{\mu }} \right)k_{2} \\ & \quad - \frac{11\sqrt 3 }{8}\left( {1 - 2\mu } \right)k_{3} + \frac{\sqrt 3 }{6}\left( {1 + \mu } \right)\delta_{1} - \frac{\sqrt 3 }{6}\left( {2 - \mu } \right)\delta_{2} , \\ \end{aligned} $$

16 $$ \Omega_{yy}^{0} = \frac{9}{4} + \frac{7}{4}\epsilon ^{\prime}\frac{3}{8}\left( {11 - \frac{7}{1 - \mu } + \frac{11\mu }{{1 - \mu }}} \right)k_{1} + \frac{3}{2\mu }k_{2} + \frac{21}{8}k_{3} + \frac{1}{2}\left( {1 - 3\mu } \right)\delta_{1} - \frac{1}{2}\left( {2 - 3\mu } \right)\delta_{2} . $$

Substituting these values into Eq. (15) and replacing $\lambda^{2}$ by $\Lambda$, the characteristic equation becomes

17 $$ \Lambda^{2} + b\Lambda + c = 0 $$

where

$$ b = 1 + 8 - 3\epsilon ^{\prime} - 3k_{1} - 3k_{2} + \frac{3}{2}k_{3} , $$

$$ \begin{aligned} c & = \frac{27\mu }{4}\left( {1 - \mu } \right) + \frac{33\mu }{2}\epsilon ^{\prime}\left( {1 - \mu } \right) + \frac{9\mu }{2}k_{1} + \frac{{9k_{2} }}{2}\left( {1 - \mu } \right) + \frac{{99\mu k_{3} }}{4}\left( {1 - \mu } \right) \\ & \quad + \frac{3\mu }{2}\left( {1 - \mu } \right)\delta_{1} + \frac{3\mu }{2}\left( {1 - \mu } \right)\delta_{2} . \\ \end{aligned} $$

Its roots

18 $$ \Lambda_{1,2} = \frac{1}{2}\left[ { - b \pm \sqrt \Delta } \right] $$

where $$\Delta = b^{2} - 4c$$ is the discriminant, Hence, the roots $$\lambda_{1} = + \Lambda_{1}^{{{\raise0.7ex\hbox{$1$} \!\mathord{\left/ {\vphantom {1 2}}\right.\kern-\nulldelimiterspace} \!\lower0.7ex\hbox{$2$}}}} ,\lambda_{2} = - \Lambda_{1}^{{{\raise0.7ex\hbox{$1$} \!\mathord{\left/ {\vphantom {1 2}}\right.\kern-\nulldelimiterspace} \!\lower0.7ex\hbox{$2$}}}} ,\lambda_{3} = + \Lambda_{2}^{{{\raise0.7ex\hbox{$1$} \!\mathord{\left/ {\vphantom {1 2}}\right.\kern-\nulldelimiterspace} \!\lower0.7ex\hbox{$2$}}}}$$ and $$\lambda_{4} = - \Lambda_{2}^{{{\raise0.7ex\hbox{$1$} \!\mathord{\left/ {\vphantom {1 2}}\right.\kern-\nulldelimiterspace} \!\lower0.7ex\hbox{$2$}}}}$$ depend on the values of the parameters $$\mu ,k_{1} ,k_{2} ,k_{3} ,\delta_{1} ,\delta_{2}$$, $$\epsilon ,$$ and $$\epsilon ^{\prime}$$. Now the discriminant can be expressed as

19 $$ \Delta = p\mu^{2} + q\mu + r $$

with

$$ p = 27\left( {1 + \frac{22}{9}\epsilon ^{\prime} + \frac{11}{3}k_{3} + \frac{2}{9}\delta_{1} + \frac{2}{9}\delta_{2} } \right) $$

$$ q = - 27\left( {1 + \frac{22}{9}\epsilon ^{\prime} + \frac{2}{3}k_{1} - \frac{2}{3}k_{2} + \frac{11}{3}k_{3} + \frac{2}{3}\delta_{1} + \frac{2}{9}\delta_{2} } \right) $$

$$ r = 1 + 16\epsilon - 6\epsilon ^{\prime} - 6k_{1} - 24k_{2} + 3k_{3} $$

Now,

$$ \frac{d\Delta }{{d\mu }} < 0,\;{\text{for}}\;0 < \mu < \frac{1}{2} $$

$$ \left( \Delta \right)_{\mu = 0} = 1 + 16\epsilon - 6\epsilon ^{\prime} - 6k_{1} - 24k_{2} + 3k_{3} > 0 $$

And

$$ \left( \Delta \right)_{{\mu = {\raise0.7ex\hbox{$1$} \!\mathord{\left/ {\vphantom {1 2}}\right.\kern-\nulldelimiterspace} \!\lower0.7ex\hbox{$2$}}}} = - \frac{23}{4} + 16\epsilon - \frac{21}{2}\epsilon ^{\prime} - 15k_{1} - 15k_{2} - \frac{87}{4}k_{3} - \frac{3}{2}\delta_{1} - \frac{3}{2}\delta_{2} < 0 $$

Since $$\Delta$$ is a strictly decreasing function of $$\mu$$ in the interval $$\left( {0,\frac{1}{2}} \right)$$ and $$\left( \Delta \right)_{\mu = 0}$$, $$\left( \Delta \right)_{{\mu = {\raise0.7ex\hbox{$1$} \!\mathord{\left/ {\vphantom {1 2}}\right.\kern-\nulldelimiterspace} \!\lower0.7ex\hbox{$2$}}}}$$ are of opposite signs, consequently, there is only one value of $$\mu$$ call $$\mu_{c}$$ in the interval $$\left( {0,\frac{1}{2}} \right)$$ for which $$\Delta$$ vanishes. This $$\mu_{c}$$ is called the critical mass ratio parameter. Therefore, we consider the following three regions of possible cases.

(i) When $0 \le \mu < \mu_{c}$, the discriminant $\left( \Delta \right)$ is positive, the values of $\Lambda_{1,2}$ given by Eq. (18) are negative and all the four characteristics roots are distinct purely imaginary. Hence, the triangular point is stable.
(ii) When $\mu = \mu_{c} ,$ the discriminant $\left( \Delta \right)$ is zero. The both values of $\Lambda_{1,2}$ given by Eq. (18) are same. These roots give secular terms in the solutions of the variational equation. Hence, the triangular point is unstable.
(iii) When $\mu_{c} < \mu \le \frac{1}{2}$, the discriminant $\left( \Delta \right)$ is negative. This shows that the real parts of the two characteristic roots are positive and equal. Hence the triangular point is unstable. Therefore we have stability for the first case and instability for the last case.

#### Critical mass

**Figure 6:**
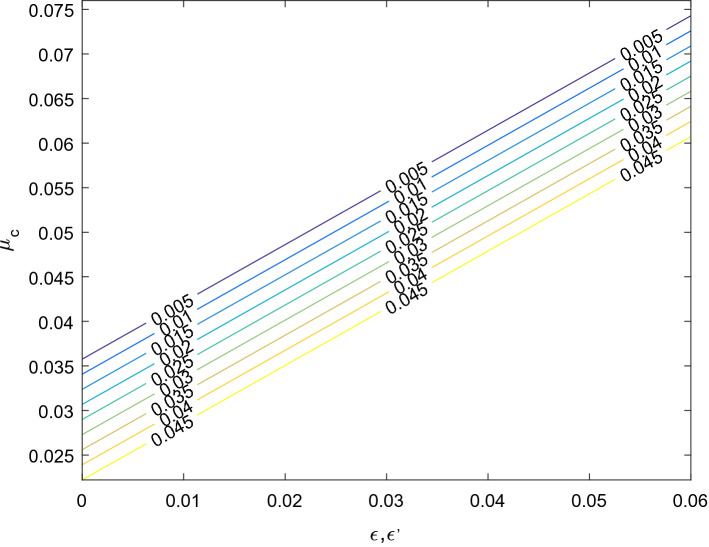
The size of the region of stability in the presence of small perturbations in Coriolis force and centrifugal force for $\delta_{1} = 0.06,\;\delta_{2} = 0.05,\;k_{1} = k_{2} = k_{3} = 0$.

**Figure 7:**
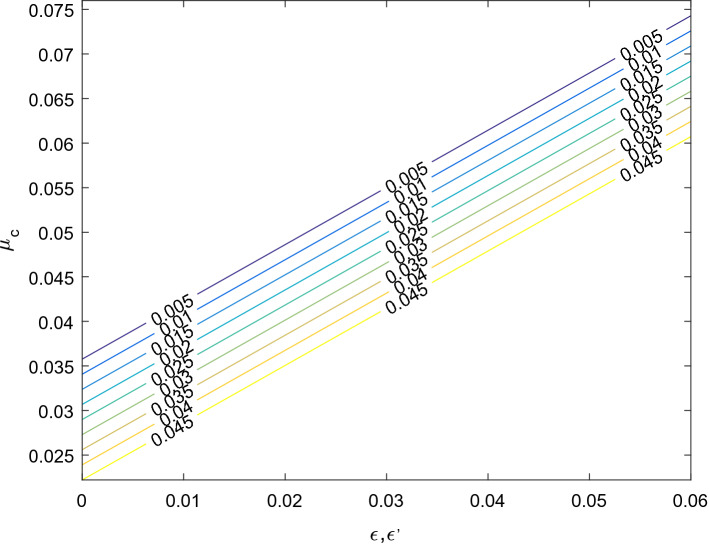
The size of the region of stability in the presence of small perturbations in Coriolis force and centrifugal force for $\delta_{1} = 0.06,\delta_{2} = 0.05,k_{1} = 1.58302 \times 10^{ - 7} ,k_{2} = 9.83933 \times 10^{ - 18} ,k_{3} = 3.13153 \times 10^{ - 8}$**.**

The solution of the equation $\Delta = 0$ obtained from (19) for $\mu$ gives the critical mass ratio value $\mu_{c}$ of the mass parameter. Then we have

20 $$ \mu_{c} = \mu_{0} + \mu_{m} + \mu_{1} + \mu_{2} + \mu_{3} + \mu_{n} $$

where

$$ \mu_{0} = \frac{1}{2}\left( {1 - \frac{{\sqrt {69} }}{9}} \right) $$

$$ \mu_{m} = \frac{4}{{27\sqrt {69} }}\left( {36\epsilon - 19\epsilon ^{\prime}} \right) $$

$$ \mu_{1} = \frac{1}{3}\left( {1 - \frac{15}{{\sqrt {69} }}} \right)k_{1} $$

$$ \mu_{2} = - \frac{1}{3}\left( {1 + \frac{15}{{\sqrt {69} }}} \right)k_{2} $$

$$ \mu_{3} = - \frac{2}{{9\sqrt {69} }}k_{3} $$

$$ \mu_{n} = - \frac{2}{{27\sqrt {69} }}(\delta_{1} + \delta_{2} ) $$

here $\mu_{c}$ represents the combined effects of small perturbations in the Coriolis and centrifugal forces, heterogeneous oblateness and radiation pressures of both primaries. However, in the absence of perturbations in the Coriolis and centrifugal forces $\mu_{c}$ agrees with the critical mass value of^7^ when both primaries are radiating and heterogeneous spheroids with three layers of different densities. In this case $\mu_{c} < \mu_{0}$, this indicates that the range of stability decreases. Further more if the primaries are neither radiating nor heterogeneous oblate spheroid, the critical mass value $\mu_{c}$ confirms the results of^2^. Also in the absence of the heterogeneous spheroids $(k_{1} = k_{2} = k_{3} = 0)$, the critical mass value $\mu_{c}$ verifies the result of^6^ when neglecting the triaxiality of the primaries $(\sigma_{1} = \sigma_{2} = \sigma_{1}^{^{\prime}} = \sigma_{2}^{^{\prime}} = 0)$. In this case $\mu_{c} > \mu_{0}$, which indicate that the range of stability increases. We observe that if the primaries are non luminous and spherical bodies and small perturbations are also absent, the critical mass value $\mu_{c}$ reduces to the classical value $\mu_{0}$ of CR3BP of the^1^. It is obvious that all perturbing parameters except $\epsilon$ have the destabilizing effects. Figures 6 and 7 shows the effects of a small perturbation in the Coriolis and centrifugal forces on the sizes of the regions of stability with and/or without the effects of heterogeneous oblateness for an arbitrary system given to the radiation pressure force. Table 7 showcase the various effects in the region of stability with the influence of the effect due heterogeneous oblate spheroids, radiation pressure, Coriolis and centrifugal forces.

**Table 7:** Effect of the parameters ($$\delta_{1} ,\delta_{2} ,$$$$\epsilon ,\epsilon ^{\prime}$$) in the stability region of equilibrium points for $$k_{1} = 1.58302 \times 10^{ - 7} ,k_{2} = 9.83933 \times 10^{ - 18} ,k_{3} = 3.13153 \times 10^{ - 8}$$.

$\delta_{1}$	$\delta_{2}$	$\epsilon$	$\epsilon ^{\prime}$	$\mu_{c}$
0	0	0	0	0.03852
0.06	0.05	0.015	0.01	0.04378
0.08	0.07	0.025	0.02	0.04646
0.10	0.09	0.035	0.03	0.04913
0.30	0.20	0.045	0.04	0.04940
0.50	0.40	0.055	0.05	0.04887
0.70	0.60	0.065	0.06	0.04833
0.90	0.80	0.075	0.07	0.04775

### Stability of collinear points

Now we consider the stability of the collinear points. At first we consider the point corresponding to *L*_*1*_.

For this point, $r_{1} = \mu - x > 1$, $r_{2} = \mu - 1 - x < 1$, $y = 0$ we have

$$ \Omega_{xx}^{o} = 1 + \epsilon ^{\prime} + \frac{3}{2}k_{3} + \left( {\frac{2(1 - \mu )}{{r_{1}^{3} }} + \frac{{6k_{1} }}{{2r_{1}^{5} }}} \right)(1 - \delta_{1} ) + \left( {\frac{2\mu }{{r_{2}^{3} }} + \frac{{6k_{2} }}{{2r_{2}^{5} }}} \right)(1 - \delta_{2} ) > 0 $$

$$ \Omega_{yy}^{0} = 1 + \epsilon ^{\prime} + \frac{3}{2}k_{3} - \left( {\frac{1 - \mu }{{r_{1}^{3} }} + \frac{{3k_{1} }}{{2r_{1}^{5} }}} \right)(1 - \delta_{1} ) - \left( {\frac{\mu }{{r_{2}^{3} }} + \frac{{3k_{2} }}{{2r_{2}^{5} }}} \right)(1 - \delta_{2} ) < 0 $$

21 $$ \Omega_{xy}^{0} = \Omega_{yx}^{0} = 0 $$

Similarly for points corresponding to *L*_*2*_ and *L*_*3*_,

$$ \Omega_{xx}^{0} > 0,\;\Omega_{yy}^{0} < 0\;{\text{and}}\;\Omega_{xy}^{0} = 0 $$

Now the characteristics Eq. (15) becomes

22 $$ \lambda^{4} + \left( {4n^{2} \alpha^{2} - \Omega_{xx}^{0} - \Omega_{yy}^{0} } \right)\lambda^{2} + \Omega_{xx}^{0} \Omega_{yy}^{0} = 0, $$

here $\Omega_{xx}^{0} \Omega_{yy}^{0} < 0$, implies that the discriminant of (22) is positive and therefore its four characteristic roots and can be expressed as

23 $$ \lambda_{1} = s,\;\lambda_{2} = - s,\;\lambda_{3} = it\;{\text{and}}\;\lambda_{4} = - it $$

where *s* and *t* are real. Thus the motion around the collinear points is unbounded therefore the collinear points are unstable. It shows that the nature of the stability of collinear points is not affected by the changes in the Coriolis and centrifugal forces, heterogeneous oblateness, or radiation pressure forces of the both primaries and they remain unstable. The Tables 3, 4 and 5 shows the behaviors of the instability of the collinear libration points $L_{1}$, $L_{2}$ and $L_{3}$.

## Discussion

The equations governing the motion of the infinitesimal body in the circular restricted three body problem with the effect of perturbations in the Coriolis and centrifugal forces when the both primaries are heterogeneous oblate spheroids with three layers of different density and the source of radiation are described in (1–3).The Eqs. (1–3) are same as in the classical case when there are no perturbations in the Coriolis and centrifugal forces and both primaries are neither heterogeneous oblate spheroid nor source of radiation pressure. The system (9) gives the positions of triangular points $L_{4,5}$. This shows that they depend on the effects of a small perturbation in the centrifugal force and heterogeneous oblateness and radiation pressures of the both primaries. They no longer form equilateral triangles with the primaries as in the classical case. Rather they form simple triangles with the primaries. The Eq. (14) differs from^7^ due to presence of small perturbations in the Coriolis and centrifugal forces. If the primaries are neither heterogeneous spheroid nor radiating and there are no perturbations in the Coriolis and centrifugal forces, the Eq. (14) represents that of the classical restricted problem. The critical value of the mass parameter $\mu_{c}$ given by Eq. (20) shows the combined effect of perturbations in the Coriolis and centrifugal forces, heterogeneous oblateness and radiation pressures of the both primaries. In the absence of all parameters ($k_{1} = k_{2} = k_{3} = \delta_{1} = \delta_{2}$ $=$ $\epsilon \epsilon ^{\prime}$) $\mu_{c}$ reduces to $\mu_{0}$ $\cong$ 0.0385208…, which corresponds to^1^. But in the absence of the effect of heterogeneous spheroids ($k_{1} = k_{2} = k_{3} = 0$) the critical mass value $\mu_{c}$ affirms the result of^6^ when there are no effects of triaxility ($\sigma_{1} = \sigma_{2} = \sigma_{1}^{^{\prime}} = \sigma_{2}^{^{\prime}} = 0$). In Table 7 the first result coincides with the^7^ and similarly with the^1^ for some certain values of the mass parameter while the other results emanate the region of stabilizing form with variation of a small perturbation in Coriolis and centrifugal forces and radiation pressure. It is observed that with the values of the parameters increase, the stability region is slightly reduced. We presented the stability of collinear equilibrium points in Tables 8, 9 and 10 according to the different cases, it is seen that no any case meet the requirement for stability of collinear points, therefore the result of the characteristics of Eq. (22) shows that the two roots are positive and negative real numbers while the other roots are positive and negative imaginary numbers. Hence it is unstable. Figures 6 and 7show that with the effect of perturbation in Coriolis and centrifugal forces the sizes of the region of stability seen to be increased. The graphs are showing that as the perturbation in centrifugal force is reducing $\mu_{C}$ is increasing, so also as the perturbation in Coriolis force increasing the $\mu_{C}$ increases.

**Table 8:** Stability of collinear point $L_{1}$.

Case	L_1_	$\Omega_{xx}^{0}$	$\Omega_{yy}^{0}$	± $\lambda_{1,2}$	± $\lambda_{3,4}$	Remark
1	− 1.20124	4.16046	− 0.58023	0.52688	2.94912*i*	Unstable
2	− 1.19837	4.21239	− 0.59120	0.53606	2.94383*i*	Unstable
3	− 1.20135	4.16431	− 0.58215	0.52798	2.94900*i*	Unstable
4	− 1.19843	4.18776	− 0.59388	0.53490	2.94826*i*	Unstable
5	− 1.18362	4.20600	− 0.60300	0.54027	2.94769*i*	Unstable
6	− 1.18647	4.18151	− 0.59075	0.53307	2.94845*i*	Unstable
7	− 1.18083	3.98648	− 0.47824	1.05279	1.31153*i*	Unstable

**Table 9:** Stability of collinear point $L_{2}$.

Case	L_2_	$\Omega_{xx}^{0}$	$\Omega_{yy}^{0}$	± $\lambda_{1,2}$	± $\lambda_{3,4}$	Remark
1	− 0.01158	16.99910	− 6.99953	3.15493	3.45746*i*	Unstable
2	− 0.01167	16.99920	− 6.99950	3.15483	3.45744*i*	Unstable
3	− 0.01176	16.99801	− 6.99540	3.15482	3.45847*i*	Unstable
4	− 0.00353	16.46300	− 6.73147	3.07469	3.42378*i*	Unstable
5	− 0.01063	15.99840	− 6.49922	3.00382	3.39467*i*	Unstable
6	− 0.01868	12.75850	− 4.87923	2.46864	3.19608*i*	Unstable
7	− 0.01071	16.00850	− 6.48922	3.63914	2.80074*i*	Unstable

**Table 10:** Stability of collinear point $L_{3}$.

Case	L_3_	$\Omega_{xx}^{0}$	$\Omega_{yy}^{0}$	± $\lambda_{1,2}$	± $\lambda_{3,4}$	Remark
1	1.19555	4.11504	− 0.55752	0.51334	2.95059*i*	Unstable
2	1.19266	4.16224	− 0.56612	0.52116	2.94542*i*	Unstable
3	1.19563	4.11505	− 0.55752	0.51335	2.95059*i*	Unstable
4	1.17871	4.13364	− 0.56682	0.51888	2.94999*i*	Unstable
5	1.17624	4.15411	− 0.57706	0.52496	2.94933*i*	Unstable
6	1.19313	4.13447	− 0.56723	0.51913	2.94996*i*	Unstable
7	1.17343	4.20187	− 0.58593	1.15636	1.35692*i*	Unstable

## Conclusion

We have investigated the location and stability of equilibrium points under the influence of small perturbations in the Coriolis and centrifugal forces when both the primaries are heterogeneous spheroids with three layers and radiating. It is found that the stability behavior of the collinear points remains unchanged despite all the perturbations involved and they are unstable. The triangular points are stable for $0 < \mu < \mu_{c}$ and unstable for $\mu_{c} \le \mu \le \frac{1}{2}$, where $\mu_{c}$ is the critical mass parameter affected by small perturbations in the Coriolis and centrifugal forces, heterogeneous oblateness and radiation pressure of both primaries. Our observation shows that all perturbations except that for Coriolis force have destabilizing tendencies and possess the decreasing size of the region of stability.
